# ProNGF, but Not NGF, Switches from Neurotrophic to Apoptotic Activity in Response to Reductions in TrkA Receptor Levels

**DOI:** 10.3390/ijms18030599

**Published:** 2017-03-09

**Authors:** Maria S. Ioannou, Margaret Fahnestock

**Affiliations:** Department of Psychiatry and Behavioural Neurosciences, McMaster University, Hamilton, ON L8S 4K1, Canada; ioannoum@janelia.hhmi.org

**Keywords:** neurotrophin, proNGF, TrkA, p75NTR, sortilin, PC12 cells, apoptosis

## Abstract

Nerve growth factor (NGF) promotes the survival and differentiation of neurons. NGF is initially synthesized as a precursor, proNGF, which is the predominant form in the central nervous system. NGF and proNGF bind to TrkA/p75NTR to mediate cell survival and to sortilin/p75NTR to promote apoptosis. The ratio of TrkA to p75NTR affects whether proNGF and mature NGF signal cell survival or apoptosis. The purpose of this study was to determine whether the loss of TrkA influences p75NTR or sortilin expression levels, and to establish whether proNGF and mature NGF have a similar ability to switch between cell survival and cell death. We systematically altered TrkA receptor levels by priming cells with NGF, using small interfering RNA, and using the mutagenized PC12nnr5 cell line. We found that both NGF and proNGF can support cell survival in cells expressing TrkA, even in the presence of p75NTR and sortilin. However, when TrkA is reduced, proNGF signals cell death, while NGF exhibits no activity. In the absence of TrkA, proNGF-induced cell death occurs, even when p75NTR and sortilin levels are reduced. These results show that proNGF can switch between neurotrophic and apoptotic activity in response to changes in TrkA receptor levels, whereas mature NGF cannot. These results also support the model that proNGF is neurotrophic under normal circumstances, but that a loss in TrkA in the presence of p75NTR and sortilin, as occurs in neurodegenerative disease or injury, shifts proNGF, but not NGF, signalling from cell survival to cell death.

## 1. Introduction

The neurotrophin nerve growth factor (NGF) affects the survival, regulation, and differentiation of both central and peripheral nervous system neurons [1]. NGF is initially translated as a precursor, proNGF, which can be cleaved intracellularly into mature NGF by furin [2], extracellularly by plasmin or matrix metalloproteinases [3,4], or remain intact and signal in its precursor form [5,6]. In the rat, mouse, and human brain, proNGF is the predominant form of NGF, whereas little to no mature NGF can be detected [7].

NGF and proNGF serve as secreted ligands for three unrelated receptors: tropomyosin-related kinase A (TrkA), p75NTR pan-neurotrophin receptor, and sortilin. TrkA receptors activate neurotrophic signaling pathways such as the phosphatidylinositol-3-kinase (PI3K)/Akt pathway and the Ras/Extracellular signal-regulated kinase (ERK) pathway [8]. The PI3K-Akt pathway is important for neurotrophin-mediated survival [9,10]. ERK is involved in signaling neuronal survival following cellular insult [10] and has an important role in neuritogenesis [11]. P75NTR is well known for its ability to induce apoptosis; however, it has also been implicated in survival signaling. In the absence of TrkA, p75NTR promotes apoptosis via the p53, ceramide, and c-Jun N-terminal kinase pathways [12]. It has been shown that p75NTR requires the co-receptor sortilin in order to induce cell death [13]. However, when co-expressed with TrkA, p75NTR increases the neurotrophin binding affinity for TrkA and enhances neuronal survival and neurite outgrowth via the NF-κB and Rho signaling pathways [14,15,16,17]. Although both NGF and proNGF are able to bind to and activate TrkA and p75NTR, they do so with different affinities. TrkA has a higher affinity for mature NGF than for proNGF [5,6]. p75NTR, on the other hand, has a higher affinity for proNGF than for mature NGF [3]. The sortilin/p75NTR complex binds to the prodomain of neurotrophins and has a high affinity for proNGF, but not NGF [13]. Consistently, NGF and proNGF are retrogradely transported in neurons with comparable kinetics; however, the majority of NGF-positive vesicles contain TrkA alone, while proNGF-positive vesicles contain both TrkA and p75NTR [18].

Several studies have demonstrated that the activity of NGF depends on the relative levels of TrkA and p75NTR. Reducing p75NTR reduces the NGF-induced survival of embryonic sensory neurons, which express both TrkA and p75NTR [19]. Rat oligodendrocytes express p75NTR and normally undergo apoptosis in response to NGF. However, NGF signals survival for TrkA-transfected oligodendrocytes [20], which shows that high levels of TrkA activation may override death signaling by p75NTR. Conversely, the overexpression of p75NTR in cortical neurons and PC12 cells, both of which produce endogenous TrkA, results in NGF-induced cell death [21], demonstrating that high levels of p75NTR may override survival signaling by TrkA. We showed that the same holds true for proNGF, and that changing the ratio of TrkA to p75NTR in primed versus unprimed PC12 cells determines whether proNGF signals survival or death [22]. However, sortilin levels were not examined in these studies. We therefore asked whether modulating TrkA levels could differentially affect levels of p75NTR and sortilin receptors, altering the outcome of proNGF versus NGF administration.

## 2. Results

### 2.1. Nerve Growth Factor (NGF) and ProNGF Support Cell Survival Similarly in Primed PC12 Cells

**Figure 1:**
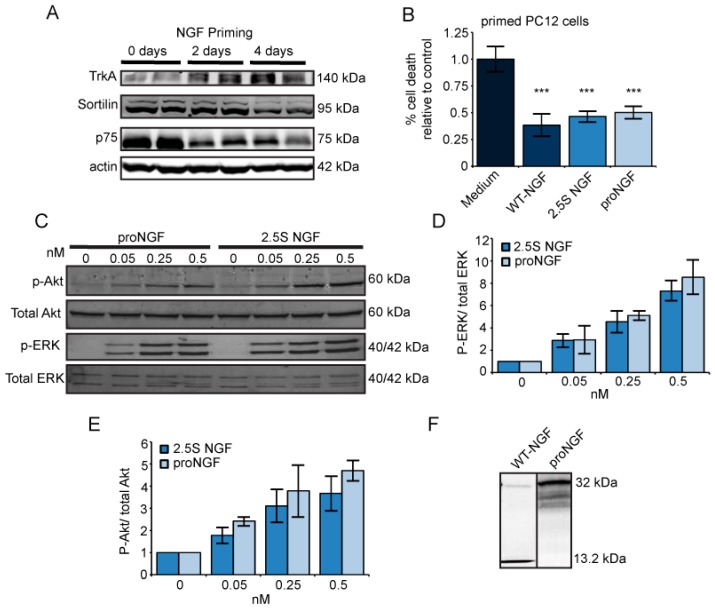
ProNGF and nerve growth factor (NGF) are neurotrophic in primed PC12 cells. (**A**) Representative Western blot of receptor levels in PC12 cells primed for 0 to 4 days with 50 ng/mL 2.5S NGF; (**B**) PC12 cells were primed for 18 h in 50 ng/mL NGF and incubated for 24 h in 0.5 nM proNGF, WT-NGF, 2.5S NGF, or medium alone. Both proNGF and NGF reduced the percentage of cell death compared to the medium alone. Percentage of cell death is expressed relative to levels in medium-treated cells. Error bars represent standard error of the mean (SEM). *** *p* < 0.001. *n* = 8 for proNGF and medium, while *n* = 7 for WT-NGF and 2.5S NGF. Data pooled from two independent experiments; (**C**) PC12 cells were primed for 18 h in 50 ng/mL NGF, treated with increasing concentrations of proNGF or NGF for 5 min, and the activation of extracellular signal-regulated kinase (ERK) and Akt was analyzed by Western blot; (**D**) Quantification of ERK activation. P-ERK is normalized to total ERK and is expressed relative to untreated cells. Error bars represent SEM. *n* = 4 for proNGF and *n* = 3 for 2.5S NGF; (**E**) Quantification of Akt activation. P-Akt is normalized to total Akt and is expressed relative to untreated cells. Error bars represent SEM. *n* = 4 for proNGF and *n* = 2 for 2.5S NGF; and (**F**) Western blot of collected media showing that proNGF remains intact throughout the experiment.

We previously reported that proNGF is apoptotic in unprimed PC12 cells with low TrkA levels, while it is neurotrophic in superior cervical ganglion (SCG) neurons and in NGF-primed PC12 cells [22]. PC12 cells differentiate into neuronal-like cells in response to NGF, and this involves changes in the expression of their receptors. Therefore, we first examined the effects of NGF priming on the expression levels of TrkA, sortilin, and p75NTR in PC12 cells (Figure 1A). Consistent with our previous report [22], priming PC12 cells with NGF resulted in an increase in TrkA levels and a decrease in p75NTR levels. Interestingly, we also found that sortilin levels decreased following NGF priming. Thus, priming PC12 cells with NGF changed the receptor profile by increasing the pro-survival receptor TrkA and decreasing the apoptotic receptors, p75NTR and sortilin.

We tested whether proNGF and mature NGF support PC12 cell survival equally well, following NGF priming. In PC12 cells primed for as little as 18 hours, treatment with WT-NGF, 2.5S NGF, and proNGF (cleavage-resistant proNGF(R-1G)) all supported cell survival, when compared to the medium alone (Figure 1B). ProNGF was as effective at supporting cell survival as WT-NGF and 2.5S NGF; 0.5 nM of each neurotrophin reduced the percentage of cell death by one-half. Furthermore, in agreement with previous reports [5,6], we found that proNGF activated the neurotrophic signaling pathways ERK and Akt in primed PC12 cells, albeit to a lesser degree than 2.5S NGF (Figure 1C–E). Although the proNGF used in these experiments was cleavage resistant, the intracellular and extracellular cleavage of proNGF to NGF has been proposed as a mechanism for proNGF neurotrophic activities [4,23]. Therefore, we collected and analyzed the medium following each experiment to verify that the proNGF remained intact (Figure 1F). Consistent with our previous report [22], proNGF and NGF support the survival of primed PC12 cells to comparable levels.

### 2.2. TrkA Knockdown Has No Effect on Cells Treated with Mature NGF but Increases Cell Death in Response to ProNGF

**Figure 2:**
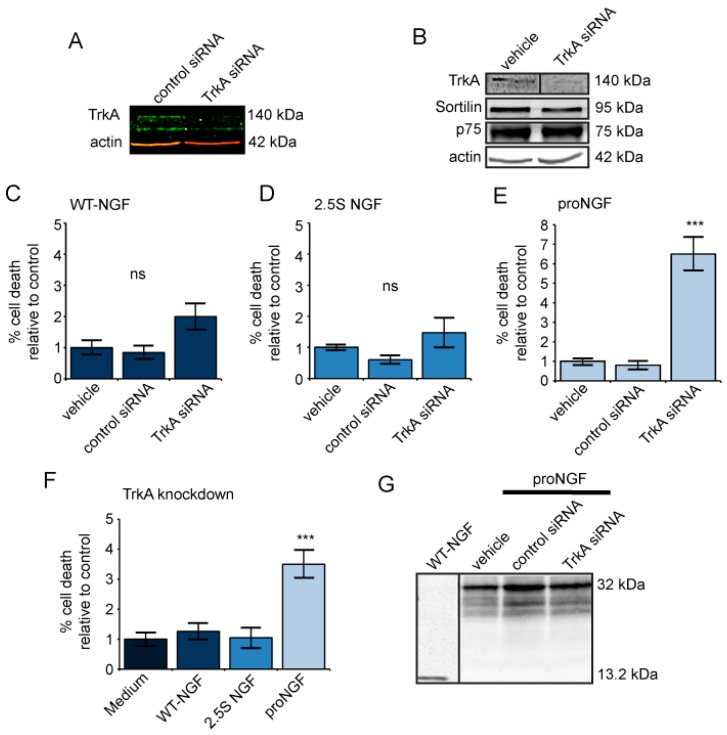
ProNGF causes cell death in primed PC12 cells when TrkA is knocked down, while mature NGF has no effect. (**A**,**B**) PC12 cells were treated with 150 nM TrkA siRNA, control siRNA, or vehicle, and primed for 18 h in 50 ng/mL 2.5S NGF. Western blot showing reduced TrkA in PC12 cells treated with TrkA siRNA, compared to control siRNA or vehicle controls. Sortilin and p75NTR levels were unchanged as a result of TrkA knockdown; (**C**–**F**) PC12 cells were transfected with 150 nM TrkA siRNA, control siRNA, or vehicle, primed for 18 h in 50 ng/mL 2.5S NGF, and treated for 24 h with 0.5 nM (**C**) WT-NGF, *n* = 11 for TrkA siRNA and control siRNA, *n* = 7 for vehicle (**D**) 2.5S NGF, *n* = 11 for TrkA siRNA, *n* = 12 for control siRNA, and *n* = 8 for vehicle (**E**) proNGF, *n* = 11 for TrkA siRNA, *n* = 12 for control siRNA, and *n* = 8 for vehicle; (**F**) PC12 cells were treated with 150 nM TrkA siRNA, primed for 18 h in 50 ng/mL 2.5S NGF, and treated for 24 h with 0.5 nM proNGF, WT-NGF, 2.5S NGF, or medium alone. *n* = 11 for proNGF, WT-NGF, and 2.5S NGF, and *n* = 12 for medium alone. Percentage of cell death is expressed relative to levels in medium-treated cells. For all graphs: Error bars represent SEM. *** *p* < 0.001. Data pooled from three independent experiments; and (**G**) Western blot of collected media showing that proNGF remained intact throughout the experiment.

We next tested whether we could abolish the neurotrophic response of proNGF in primed PC12 cells by using siRNA to knock down TrkA. Western blotting revealed a decrease in TrkA expression, but no change in sortilin or p75NTR levels (Figure 2A,B). In the absence of TrkA, WT-NGF and 2.5S NGF had no effect on cell survival in primed PC12 cells, compared to vehicle or non-targeting siRNA (Figure 2C,D). ProNGF, on the other hand, not only failed to support cell survival, but instead induced apoptosis when TrkA was reduced. Following proNGF treatment, the mean percentage of cell death was six times greater when TrkA was reduced using siRNA, compared to the controls (Figure 2E). There was no difference in the level of proNGF-induced cell death between non-specific siRNA or vehicle, indicating that the increase in cell death was due to the reduction of TrkA and not due to the non-specific effects of siRNA or the transfection procedure (Figure 2E). There was approximately three times the amount of cell death when cells treated with proNGF were compared to WT-NGF, 2.5S NGF, or the medium alone (Figure 2F). Finally, Western blotting of conditioned medium demonstrated that the cleavage-resistant proNGF remained intact during the experiment (Figure 2G). Therefore, proNGF is more sensitive to changes in receptor levels than mature NGF.

### 2.3. ProNGF, but Not NGF, Induces Cell Death in Mutagenized PC12s That Lack TrkA

**Figure 3:**
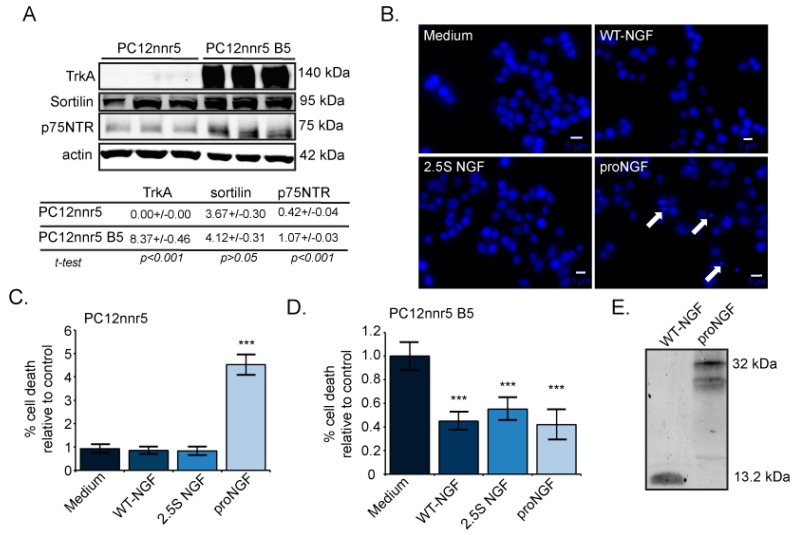
ProNGF is apoptotic in PC12nnr5 cells lacking TrkA. (**A**) Top panel: Representative Western blot of primed PC12nnr5 and PC12nnr5 B5 cell lysates. Each lane is derived from a separate plate. Bottom panel: quantification of A, where relative amounts of TrkA, sortilin, and p75NTR were normalized to β-actin. Similar results were obtained in a second experiment; (**B**) PC12nnr5 cells were treated for 48 h with medium alone or 0.5 nM WT-NGF, 2.5S NGF, or proNGF. White arrows show representative dead cells. Scale bars represent 5 μm; (**C**) ProNGF causes more apoptosis than medium, WT-NGF, or 2.5S NGF in PC12nnr5 cells. *n* = 10 for all groups. Data pooled from two independent experiments; (**D**) Re-expression of TrkA in PC12nnr5 B5 cells treated for 48 h with 0.5 nM proNGF, WT-NGF, or medium alone. *n* = 14 for proNGF and WT-NGF and *n* = 15 for 2.5S and medium alone. Data pooled from three independent experiments. Percentage of cell death is expressed relative to levels in medium-treated cells. Error bars represent SEM. *** *p* < 0.005; and (**E**) Western blot of collected media showing that proNGF remained intact throughout the experiment.

We next sought to confirm our finding that proNGF is more sensitive than NGF to changes in receptor levels, by taking advantage of the PC12nnr5 cell line. PC12nnr5 is a mutagenized cell line known for its non-responsiveness to NGF, as it has a reduced expression of both TrkA and p75NTR. Additionally, rescue experiments can be performed using the PC12nnr5 B5 cell line, in which TrkA has been stably reintroduced [24,25]. We first determined the receptor profiles of these two cell lines at the protein level using Western blot analysis (Figure 3A top panel). The mean densitometric value for each receptor was normalized to the mean densitometric value for β-actin (Figure 3A bottom panel). As expected, we found undetectable levels of TrkA protein in PC12nnr5 cells, while PC12nnr5 B5 cells expressed high levels of TrkA. Sortilin levels trended slightly higher in PC12nnr5 B5 than in PC12nnr5 cells, but did not differ significantly between the two cell lines. Interestingly, the p75NTR protein levels in PC12nnr5 B5 cells were expressed at more than twice the amount as in PC12nnr5 cells.

We then tested the response of PC12nnr5 cells to pro- and mature NGF. When exposed to proNGF, PC12nnr5 cells underwent cell death (Figure 3B,C). ProNGF induced over four times more cell death than WT-NGF, 2.5S NGF, or the medium alone. However, there was no difference in cell death between untreated cells and those treated with WT-NGF or 2.5S NGF (Figure 3C). When TrkA was re-expressed in PC12nnr5 B5 cells, proNGF, WT-NGF, and 2.5S NGF all exhibited neurotrophic activity (Figure 3D). All neurotrophin treatments reduced cell death by more than one-half, compared to cells treated with the medium alone. Western blotting of conditioned medium demonstrated that cleavage-resistant proNGF remained intact during the experiment (Figure 3E). These results confirm that proNGF is more sensitive to changes in receptor levels than NGF.

## 3. Discussion

The biological activity of proNGF has been the subject of much debate, as it has been reported to exhibit both neurotrophic [5,6,22] and apoptotic activity [3,13]. There is a great need to reconcile these conflicting studies to fully understand the function of proNGF. We have previously shown that proNGF may signal differently, depending on the culture conditions used, suggesting that this is due to the relative levels of its receptors [22]. In this study, we systematically modulated TrkA levels in PC12 cells and found that, as expected, proNGF activity depends upon the relative levels of its receptors, TrkA and p75NTR/sortilin. Moreover, we discovered that proNGF is more sensitive to changes in receptor levels than mature NGF; that is, proNGF, but not NGF, switches between neurotrophic and apoptotic activity in response to changes in receptor levels.

We modulated the receptor ratio by priming PC12 cells with NGF, reducing TrkA in primed PC12 cells using siRNA and by using the PC12nnr5 cell line. We found that, while both proNGF and NGF could support the survival of primed PC12 cells expressing relatively high amounts of TrkA, proNGF, but not NGF, induced cell death when TrkA levels were reduced using siRNA or when TrkA was eliminated, as in PC12nnr5 cells. This is consistent with Pagadala et al. [26], who showed that treatment with proNGF was able to induce cell death in PC12nnr5 cells. Furthermore, the re-expression of TrkA in PC12nnr5 cells recovered the ability of NGF to induce differentiation [27]. Here, we show that the re-expression of TrkA also rescues the neurotrophic cell survival activity of proNGF.

Importantly, we show that proNGF is more sensitive to disturbances in receptor levels than NGF. In primed PC12 cells with high TrkA levels relative to p75NTR, both NGF and proNGF signal survival. However, reduced TrkA renders the cells apoptotic in response to proNGF treatment, but non-responsive to NGF. This non-responsiveness is consistent with the report that NGF-induced survival was reduced to control levels when TrkA was inhibited using the drug K252a [28]. We found that, unlike NGF, proNGF not only fails to support survival in the absence of TrkA, but also signals cell death. This can be explained by the different receptor binding affinities of NGF and proNGF. ProNGF binds to p75NTR with a five-fold greater affinity than NGF [3], while NGF binds to TrkA with a four-fold greater affinity than proNGF [5].

The findings presented here may explain several inconsistencies in the proNGF literature, where different cell culture systems could affect proNGF activity. In dissociated SCG neurons, a commonly used cellular model, TrkA and p75NTR levels change with age. At birth, the ratio of TrkA to p75NTR mRNA in murine SCG neurons is approximately three to one, but by postnatal day four, the ratio has already dropped to less than two to one [29]. Intriguingly, proNGF is neurotrophic in postnatal day one neurons [5,22], but apoptotic in postnatal day one to three neurons maintained in culture for an additional five days [13]. Therefore, the age of the neurons or other conditions which modulate receptor levels may determine the outcome of the response to proNGF.

Our data also support the model that sortilin levels modulate proNGF signaling. ProNGF forms a complex with p75NTR and its co-receptor sortilin, while mature NGF does not interact with sortilin [13]. Here, we find that proNGF signals cell death in unprimed PC12 cells and PC12nnr5 cells, where sortilin levels are higher, and signals survival in primed PC12 cells where sortilin levels decrease. In fact, proNGF transitions from neurotrophic to apoptotic, as responsive neurons age; knocking out or blocking sortilin reduces cell death in older basal forebrain and SCG neurons [30,31]. Thus, modulating sortilin levels can also determine the physiological response of cells treated with proNGF.

A reduced ratio of TrkA to p75NTR/sortilin in the presence of elevated proNGF could be ultimately responsible for neuronal cell death in Alzheimer’s disease (AD) and CNS injury. In the early stages of AD, there is a reduction in cortical TrkA but no change in p75NTR or sortilin [32,33]. ProNGF levels increase in cortical and hippocampal tissues and correlate with cognitive decline [7,34,35]. Following spinal cord injury, secondary cell death in oligodendrocytes is caused by the upregulation of p75NTR in the presence of increased proNGF surrounding the lesion center [36]. In fact, the delivery of small molecules to block the binding of proNGF to p75NTR following spinal cord injury, promotes functional recovery by increasing the number of surviving oligodendrocytes and myelinated axons [37]. Following lesions of the internal capsule, increased sortilin in corticospinal neurons is responsible for neuronal cell death [31]. This suggests that TrkA, p75NTR, and sortilin levels are balanced in a way that normally allows proNGF to support cell survival. On the other hand, a reduction in TrkA or an increase in p75NTR and/or sortilin may result in a receptor ratio that favours the signalling of cell death by proNGF.

Alternatively, an increased ratio of TrkA to p75NTR/sortilin in the presence of proNGF could promote cancer progression. Increasing evidence points to NGF and proNGF as diagnostic markers of both thyroid and prostate cancer [38,39]. NGF and proNGF are expressed by cancer cells and mediate cancer-related phenomena, such as angiogenesis and pain. For example, endothelial cells respond to NGF by enhancing blood vessel formation [40,41]. In fact, the overexpression of TrkA alone is sufficient to promote angiogenesis in mouse models of breast cancer [42]. NGF and proNGF also mediate cancer pain through activation of TrkA on sensory neurons, and this pain can be ameliorated using TrkA inhibitors [43]. Furthermore, cancer cells respond directly to NGF and proNGF, to exacerbate cancer-related phenotypes [44]. For example, proNGF can stimulate the invasion of melanoma and breast cancer cells through the activation of p75NTR/sortilin and TrkA/sortilin, respectively [44,45,46]. In fact, an increased expression of TrkA, as observed in breast cancer, is associated with enhanced growth and metastasis [47]. Thus, given the sensitivity of proNGF to alterations in TrkA that we discovered here, it seems that in cancer, where TrkA levels are elevated, proNGF may promote angiogenesis and cancer pain, and support the survival of cancer cells when it otherwise would not.

Taken together, our results confirm that proNGF signals survival or death, depending on the relative levels of its receptors: TrkA, p75NTR, and sortilin. We show that proNGF and NGF signal differently, depending on the relative receptor levels, and that proNGF is more sensitive than NGF to disturbances in receptor levels, signaling apoptosis in conditions where NGF does not. These findings help explain the conflicting activities reported for proNGF and provide insight into the biological activity of proNGF that is essential for understanding the role of proNGF in healthy neurons and in disease.

## 4. Materials and Methods

### 4.1. Cell Culture

PC12 (rat pheochromocytoma) (ATCC, Manassas, VA, USA) cells were grown in Roswell Park Memorial Institute medium (RPMI) (Invitrogen, Burlington, ON, Canada) supplemented with 10% horse serum, 5% fetal bovine serum, and 1% penicillin/streptomycin. PC12nnr5 cells are a line of mutant PC12 cells selected for their non-responsiveness to NGF [24]. PC12nnr5 B5 cells have been stably transfected to express TrkA [25]. PC12nnr5 cells and PC12nnr5 B5 cells were received as a gift from Susan O. Meakin (The University of Western Ontario, London, ON, Canada). Both cell lines were grown in Dulbecco’s Modified Eagle’s Medium (DMEM) (Invitrogen, Burlington, ON, Canada) supplemented with 5% fetal bovine serum, 5% horse serum, 1% l-glutamine, and 1% penicillin/streptomycin. A total of 100 µg/mL of G418 Geneticin^®^ (Sigma-Aldrich, Oakville, ON, Canada) reagent was added as a selection reagent to the medium of PC12nnr5 B5 cells, to maintain stable transfectants. All cells were incubated at 37 °C at 5% CO_2_. All cell culture media and additives were obtained from Invitrogen (Burlington, ON, Canada).

### 4.2. Neurotrophins

Experiments were performed using 2.5S NGF purified from mouse salivary glands [7,48], wild type, cleavable proNGF (WT-NGF), and cleavage-resistant proNGF(R-1G) expressed in an insect cell expression system [5]. WT-NGF was cleaved almost entirely to the mature form (Figure 1D) and served as a control for insect cell expression.

### 4.3. Western Blotting

Cells were lysed in 50 mM Tris-HCl, pH 7.4, 1% NP-40, 0.25% sodium deoxycholate, and 150 mM NaCl, containing an EDTA-free protease inhibitor tablet (Roche, Mississauga, ON, Canada), 1 mM EGTA, 1 mM Na_3_VO_4_, 1 mM sodium pyrophosphate, 1 μg/mL aprotinin, and 1 μg/mL pepstatin. The protein concentrations of lysates were determined using the DC protein assay (Bio-Rad Laboratories, Hercules, CA, USA), and 25 µg total protein was loaded per lane. Western blotting was performed using affinity-purified rabbit polyclonal NGF antibody (MC-51), anti-TrkA rabbit polyclonal antibody (Chemicon, Mississauga, ON, Canada), anti-sortilin rabbit monoclonal antibody (Alomone Labs Ltd., Jerusalem, Israel), anti-p75NTR rabbit polyclonal antibody (Upstate Biotechnology Inc., Lake Placid, NY, USA), anti-phosphorylated anti-extracellular signal-regulated kinase (ERK) 1/2, Thr202/Tyr204), and total ERK (Cell Signaling Technology, Inc., Mississauga, ON, Canada), anti-phosphorylated Akt (Ser473) and total Akt, or monoclonal mouse anti-β-actin antibody (clone AC-51, Sigma, Oakville, ON, Canada). The secondary antibodies used were IRDye^®^ 680 conjugated goat polyclonal anti-rabbit IgG or IRDye^®^ 800 conjugated polyclonal anti-mouse IgG (LI-COR Biosciences, Lincoln, NE, USA). The membranes were scanned at 680 nm and/or 800 nm using the Odyssey Infrared Imaging System (LI-COR Biosciences, Lincoln, NE, USA).

### 4.4. TrkA Knockdown Bioassay

siRNA targeting rat TrkA (Qiagen, Mississauga, ON, Canada) was custom designed according to Bumeister et al. [49] (Sense: r(AUGUGGACAGAGGAGCAAA)dTdT Antisense: r(UUUGCUCCUCUGUCCACAU)dTdT), and contained a 3′-AlexaFluor647 modification on the sense strand. AllStars Negative siRNA with a 3′-AlexaFluor488 modification on the sense strand (Qiagen) was used as a non-targeting negative control. One day prior to transfection, PC12 cells were plated at 17,000 cells per well in RPMI, 10% horse serum, and 5% fetal bovine serum on Cell+ 96 well plates (Sarstedt, Montreal, QC, Canada). Cells were transfected for six hours with 150 nM TrkA siRNA or 150 nM of non-specific siRNA using Lipofectamine™ 2000 reagent (Invitrogen), according to the manufacturer’s protocol, after which time the medium was replaced with growth medium containing 50 ng/mL 2.5S NGF. Cells treated with Lipofectamine in the absence of any siRNAs are referred to as the vehicle control. 24 h following transfection, cells were washed in RPMI and treated with 0.5 nM proNGF, WT-NGF, 2.5S NGF, or medium alone without serum for 24 or 48 h.

### 4.5. PC12nnr5 Bioassay

PC12nnr5 and PC12nnr5 B5 cells were plated at 1.5 × 10^4^ and 1.2 × 10^4^ cells per well, respectively, on a Sarstedt Cell+ 96-well plate the day before the experiment. The cells were washed once with RPMI and treated with 0.5 nM proNGF, 2.5S NGF, WT-NGF, or medium alone without serum for 48 h.

### 4.6. Analysis of Cell Death

Cells were fixed with 4% paraformaldehyde, permeabilized in 0.1% Triton X-100, 0.1% sodium citrate, and stained with 1 μg/mL Hoechst (bis benzimide H33342 trihydrochloride, Sigma-Aldrich) in PBS for 5–15 min at room temperature. Images were captured using a high-resolution widefield fluorescent microscope (Leica DMI 3000B, Concord, ON, Canada) at 20× magnification, attached to a Hamamatsu Orca ER-AG camera (Bridgewater, NJ, USA). The acquisition software used was Volocity 4 (Improvision, Waltham, MA, USA) and 377/50, 472/30 and 628/40 filters were used for Hoechst, non-specific siRNA, and TrkA siRNA, respectively. For siRNA experiments, only cells with visible siRNA in the cell body were counted. Approximately 50 cells per well were counted for cells treated with TrkA siRNA or non-specific siRNA, and approximately 500 cells per well for vehicle or no treatment. For PC12nnr5 and PC12nnr5 B5 experiments, approximately 500 cells per well were counted from two independent experiments. Cells with Hoechst-stained nuclei that appeared dense and fragmented were counted as dead cells.

### 4.7. Statistical Analysis

Statistical analysis was performed using PASW Statistics 17 (SPSS, Chicago, IL, USA) software. Between-group differences were analyzed using unpaired *t*-tests or one-way analysis of variance with a post hoc Dunnett’s test.

## Abbreviations

DMEM	Dulbecco’s Modified Eagle’s Medium
EDTA	Ethylene diamine tetra acetic acid
ERK	Extracellular signal-regulated kinase
Hoechst	Bis benzimide H33342 trihydrochloride
NGF	Nerve growth factor
p75NTR	Pan-neurotrophin receptor
PBS	Phosphate buffered saline
PC12	Rat pheochromocytoma
PI3K	Phosphatidylinositol-3-kinase
proNGF	Precursor to nerve growth factor
proNGF(R-1G)	Cleavage-resistant proNGF
RPMI	Roswell Park Memorial Institute medium
SCG	Superior cervical ganglion
siRNA	Small interfering RNA
TrkA	Tropomyosin-related kinase A
WT-NGF	Cleavable, wild type proNGF
